# Dysarthria as a Presenting Symptom With Rapidly Progressive Imaging Features in Sporadic Creutzfeldt-Jakob Disease: A Case Report

**DOI:** 10.7759/cureus.62687

**Published:** 2024-06-19

**Authors:** Tengwei Pan, Shanshan Wang

**Affiliations:** 1 Neurology, Taizhou Hospital of Zhejiang Province Affiliated to Wenzhou Medical University, Taizhou, CHN

**Keywords:** unusual clinical manifestations, diffusion weighted imaging (dwi), tau- protein, prion protein, sporadic creutzfeldt-jakob disease

## Abstract

Sporadic Creutzfeldt-Jakob disease (sCJD) is a rare and fatal neurodegenerative disorder belonging to a group of diseases known as prion disease. Characterized by the formation of abnormal prion proteins in the brain, these conditions lead to tissue damage and vacuolation, giving the brain a sponge-like appearance. sCJD represents the most prevalent form of CJD, accounting for roughly 85% of all CJD cases. We report a case with unusual clinical manifestations. The patient experienced progressive neurological symptoms and MRI progression.

## Introduction

Sporadic Creutzfeldt-Jakob disease (sCJD) is a rapidly progressing neurodegenerative syndrome characterized by the accumulation of abnormally folded prion proteins in the brain. Its incidence stands at approximately one per million, with an exceedingly diverse clinical presentation. Common manifestations include rapidly progressive dementia, cerebellar ataxia, and myoclonus [1]. Atypical cases, initially presenting with focal neurological deficits, can make diagnosis particularly challenging. Isolated speech impairment as the primary manifestation of sCJD is notably rare, occurring in roughly 1% of patients [2]. This report documents a case of sCJD where isolated speech impairment was the initial symptom, accompanied by swiftly evolving radiological findings.

## Case presentation

A 56-year-old male patient was admitted on December 30, 2023 with a chief complaint of speech impairment that had persisted for two weeks. The onset of slurred speech occurred 14 days prior to hospitalization, without accompanying limb weakness, consciousness impairment, or seizures. Notably, seven days before admission, his speech difficulties significantly worsened, severely hindering effective communication. A neurological examination confirmed the presence of slurred speech, while the remainder of the physical examination was unremarkable.

The patient demonstrated intact cognitive function during the intelligence assessment, as evidenced by a Mini-Mental State Examination score of 30/30.

Comprehensive laboratory evaluations were conducted on the same day, December 30, 2023, including a complete blood count, coagulation profile, liver and renal function tests, glucose, lipid profile, electrolyte levels, and thyroid function tests, all of which returned results within normal ranges, suggesting no overt systemic abnormalities.

An EEG performed on admission revealed moderately abnormal patterns, characterized by diffuse high-amplitude slow-wave activity.

**Figure 1:**
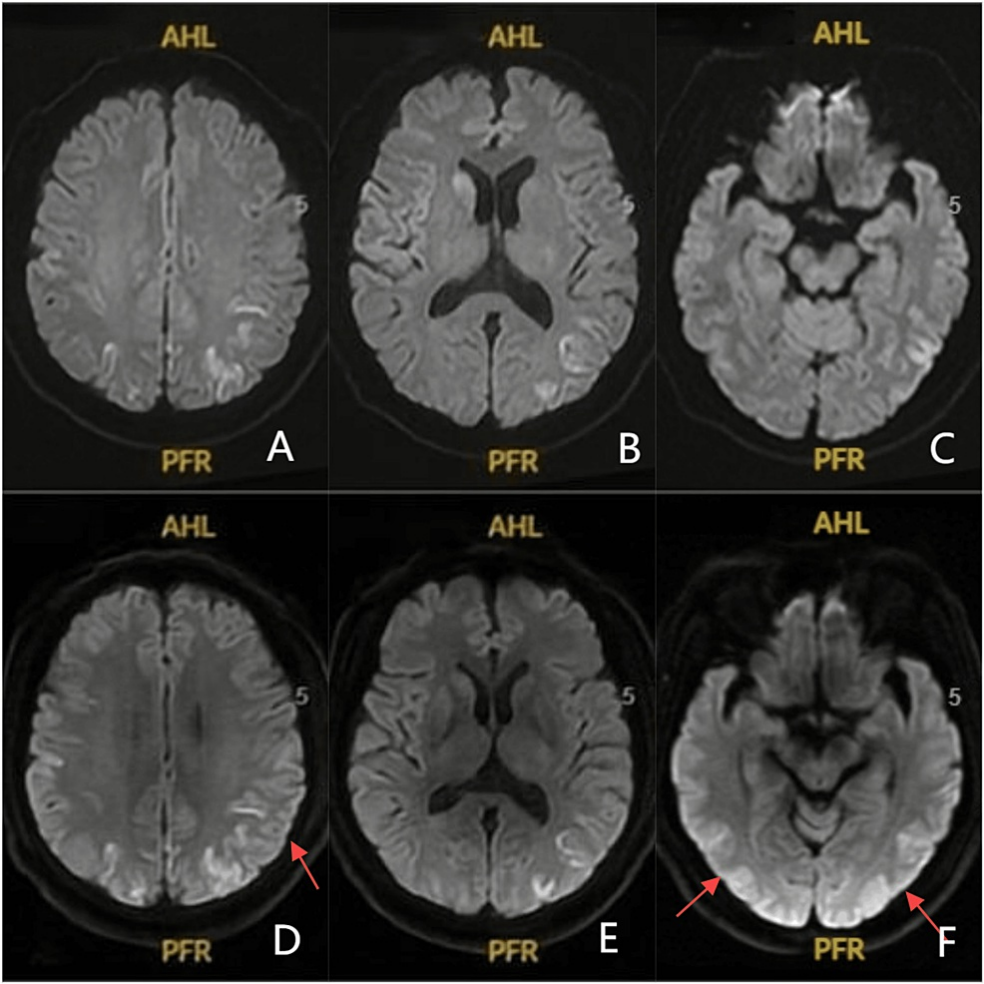
MRI of the head conducted on December 30, 2023 (A, B, and C) and January 1, 2024 (D, E, and F) A, B, and C display the DWI sequences from the MRI of the head conducted on December 30, 2023. These images depict bilateral cortical hyperintensities in a distinctive ribbon-like pattern. D, E, and F illustrate the follow-up DWI sequences from the MRI of the head performed on January 1, 2024. These images reveal a marked advancement in the extent and intensity of the abnormal high signals within the cerebral cortices. DWI, diffusion-weighted imaging

Diffusion-weighted imaging (DWI) of the brain disclosed conspicuous abnormalities, manifesting as high signal intensities in a distinctive ribbon-like pattern across both cerebral cortices. A follow-up DWI on January 1, 2024 depicted a pronounced progression of these abnormalities (Figure 1).

The results of the CSF analysis are detailed in Table 1. The level of total tau (t-tau) in the CSF was 4,921.27 pg/mL (reference range: <399 pg/mL), while phosphorylated tau 181 was 93.33 pg/mL (reference range: <50 pg/mL).

**Table 1:** CSF analysis results

Parameter	Value	Reference range
CSF color	Colorless	Colorless
CSF appearance	Clear	Clear
Proteins	290 mg/dL	200-400 mg/dL
Total cell account	1 × 10^6^/L	0-8 × 10^6^/L
Glucose	4.14 mmol/L	2.22-3.89 mmol/L
Paraneoplastic syndrome-associated antibodies	Negative	Negative
Autoimmune encephalitis-associated antibodies	Negative	Negative
Total tau	4,921.27 pg/mL	<399 pg/mL
Phosphorylated tau 181	93.33 pg/mL	<50 pg/mL

Therapeutic intervention and disease evolution

Following hospital admission, the patient received symptomatic management. Despite this, the clinical symptoms deteriorated, marked by a worsening of dysarthria and the gradual emergence of ataxia leading to loss of independent ambulation, along with deteriorating memory and diminished capacity for calculation. On January 11, the patient discharged himself against medical advice, already requiring assistance to ambulate.

A post-discharge follow-up conducted one month later documented a profound decline in cognitive function. The patient had become bedridden, incapable of self-care, and began experiencing recurrent episodes of epileptic seizures. Tragically, a telephonic assessment conducted three months post-discharge revealed that the patient had been admitted to the ICU, requiring continuous sedation and ventilator support.

## Discussion

CJD, the most prevalent human prion disease, is a fatal neurodegenerative condition causing subacute and progressive deterioration of cognitive, motor, and behavioral functions over weeks to months. CJD is categorized into sporadic, variant, familial, and iatrogenic forms based on etiology. While no effective therapy exists, early recognition and diagnosis of CJD are crucial for preventing its transmission, even though they pose clinical challenges due to the disease’s atypical manifestations and lack of early diagnostic and therapeutic options.

sCJD, accounting for nearly 85% of cases, typically presents with rapid cognitive decline, ataxia, and myoclonus [1]. Less common inaugural symptoms include psychiatric disturbances, visual impairments, seizures, and speech disorders [3]. These atypical presentations can lead to misdiagnosis or underdiagnosis. In this case, the patient initially presented with isolated speech disturbance, which later developed characteristic sCJD symptoms such as ataxia and cognitive impairment, corroborated by MRI and CSF analyses.

Brain biopsy, the gold standard for CJD diagnosis, is invasive and carries risks related to disease transmission. Therefore, MRI of the head and CSF examination are preferred diagnostic tools. MRI, being highly sensitive and specific, noninvasive, and widely available, is invaluable for sCJD assessment. About 80% of sCJD cases exhibit typical MRI findings, including hyperintensities in the caudate nucleus, putamen, or cerebral cortex on DWI and FLAIR sequences [4,5]. A recent meta-analysis found DWI to have a sensitivity of 91% and a specificity of 97% in early sCJD diagnosis [6]. The patient’s MRI showed multifocal DWI hyperintensities in the occipital lobe and basal ganglia, consistent with other sCJD reports. The 2009 WHO diagnostic criteria for CJD incorporated typically restricted diffusion patterns on DWI and high signal intensity on FLAIR images [5]. The rapid progression seen in this patient’s MRI scans over two days, although rarely reported in the literature for sCJD, underscores the significance of serial imaging in cases with initially atypical MRI findings.

Tau, a microtubule-associated protein expressed in neurons and glial cells, has been proposed as a diagnostic biomarker for sCJD in CSF, with studies reporting sensitivities and specificities around 90% [7]. However, consensus on the optimal t-tau threshold for diagnosing sCJD is lacking, with varying cutoffs suggested across studies. Elevated CSF t-tau levels in this patient (4,921.27 pg/mL) far exceeded various diagnostic thresholds proposed (e.g., >1,072 pg/mL, >1,250 pg/mL, >1,300 pg/mL, and >1,400 pg/mL) [8], strongly supporting the sCJD diagnosis. Moreover, high t-tau levels have been correlated with disease prognosis [9], suggesting that patients with elevated t-tau might experience a more rapid course of illness, as observed in our case with the patient requiring life-sustaining interventions within three months of discharge.

## Conclusions

Atypical presenting symptoms often lead to delayed or altogether overlooked diagnoses of sCJD. Nonetheless, rapidly progressive dementia and myoclonus should raise suspicion for sCJD. In recent times, advancements in MRI techniques and the identification of specific CSF biomarkers have proven instrumental in facilitating earlier detection of sCJD.
